# Toxoplasmose oculaire atypique chez une femme congolaise de 72 ans: à propos d'une observation

**DOI:** 10.11604/pamj.2015.22.267.8137

**Published:** 2015-11-20

**Authors:** Yogolelo Asani Bienvenu, Musau Nkola Angel, Kasamba Ilunga Eric, Kapalu Mwangala Socrate, Mbuyi Musanzayi Sebastien, Cilundika Mulenga Philippe, Kabamba Ngombe Leon, Iye Ombamba Kayimba Bruno, Chenge Borasisi Gaby

**Affiliations:** 1 Université de Lubumbashi, Faculté de Médecine, Service d'Ophtalmologie, République Démocratique du Congo; 2 Université de Lubumbashi, Faculté de Médecine, Département de Santé Publique, République Démocratique du Congo; 3 Université de Lubumbashi, Faculté de Médecine, Département de Biologie Moléculaire, Service de Laboratoire, République Démocratique du Congo; 4 Centre Ophtalmologique Sainte Bernadette, République Démocratique du Congo; 5 Université de Lubumbashi, Faculté de Médecine, Département de Chirurgie, République Démocratique du Congo; 6 Université de Kamina, Faculté de Médecine, Département de Sante publique, Unité de toxicologie, République Démocratique du Congo

**Keywords:** Toxoplasmose oculaire, rétinite pigmentaire, adulte, congolais, ocular toxoplasmosis, retinitis pigmentosa, adult, Congolese

## Abstract

Les auteurs rapportent un cas de toxoplasmose oculaire binoculaire, rarement décrit dans la littérature, chez une personne âgée de 72 ans, de sexe féminin, à laquelle s'associe une rétinite pigmentaire unilatérale. Cette observation permet d'attirer l'attention de la communauté scientifique sur les autres formes ou variétés de présentation moins courantes, « atypiques », pouvant être rencontrées ou associées à la toxoplasmose oculaire.

## Introduction

La toxoplasmose est une maladie habituelle chez les oiseaux et les mammifères. Elle est causée par un protozoaire obligatoirement intracellulaire appelé Toxoplasma Gondii. Helenor Campbell Wilder a identifié la présence de Toxoplasma Gondii dans l’œil [1]. La toxoplasmose oculaire est une affection très fréquente et généralement bénigne. Elle entraine de graves complications chez la femme enceinte et donne une fœtopathie chez l'immunodéprimé. Une nouvelle idée permet d’évoquer la gravité de certaines toxoplasmoses acquises qui peuvent parfois donner des lésions oculaires [2]. Dans le monde, il existe de nombreuses études menées sur ce sujet fournissant une compréhension meilleure des lésions oculaires sur le plan épidémio-clinique, paraclinique, thérapeutique et évolutif. Dans notre milieu, il n'y a pas des données relatives à cette pathologie. Le but de ce travail est de décrire cette pathologie chez une patiente âgée de 72 ans, congolaise de race noire, et d'attirer l'attention de la communauté scientifique sur les autres formes de présentation moins courantes, « atypiques » de la toxoplasmose oculaire.

## Patient et observation

**Figure 1:**
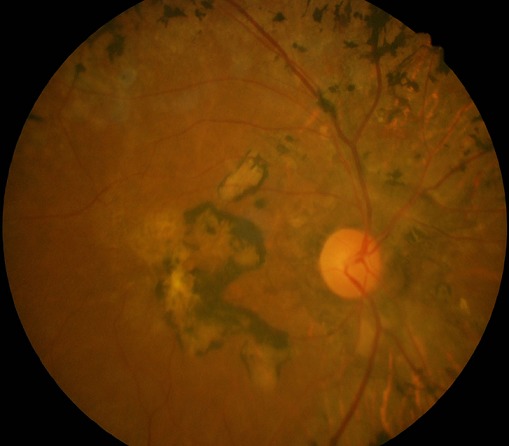
Grosse cicatrice choriorétinienne maculaire avec présence d'ostéoblastes dans la périphérie rétinienne à l’œil droit

**Figure 2:**
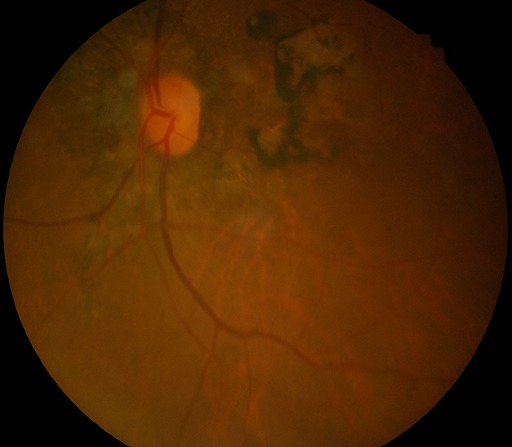
Cicatrice choriorétinienne maculaire œil gauche

Nous présentons dans ce travail le cas d'une patiente âgée de 72 ans, qui a été consultée le service d'ophtalmologie(CUL) pour une baisse de vision de loin constatée il y a environ une année, de survenue brusque. Le traitement appliqué a été à base de collyre dont elle ignore le nom. Dans les antécédents socioprofessionnels, la patiente nous a signalé qu'elle était éleveur des poules et grande consommatrice de viande. L'acuité visuelle sans correction de loin aux deux yeux était de mouvement mains à 30 cm, non améliorable par les verres correcteurs; l'acuité visuelle avec correction de près était non significative, car elle lit difficilement P4. L'examen à la lampe à fente a retrouvé une opacité corticale antérieure débutante aux deux yeux. La tonométrie par aplanation était de 17 mmHg à l’œil droit et 18 mmHg à l’œil gauche. L'examen du fond d’œil a révélé une grosse cicatrice choriorétinienne maculaire beaucoup plus marquée à l’œil droit qu’à l’œil gauche et faisant penser à une cicatrice toxoplasmique et la présence d'ostéoblastes dans la région rétinienne périphérique de l’œil droit, typique de la rétinite pigmentaire(Figure 1, Figure 2). Un examen complémentaire a été réalisé dans le cadre d'un bilan étiologique: les tests Elisa Toxoplasma IgG à 189 et Elisa Toxoplasma IgM à 56 suggérant une toxoplasmose oculaire récemment acquise et leur avidité était supérieure à 40% (67%), suggérant une infection à Toxoplasma Gondii.

## Discussion

La toxoplasmose oculaire est une maladie récurrente se développant progressivement et menaçant la fonction visuelle [3]. Toxoplasma Gondii est un parasite. Il se multiplie dans l'intestin des félidés qui libèrent des oocytes dans leurs fèces. La consommation de viande ou d'organes contenant des kystes peuvent entrainer l'apparition d'une toxoplasmose dans de nombreuses espèces, y compris le chat et l'homme. Contrairement à une idée reçue, le risque qu'un chat soit source du parasite pour l'homme est faible [4]. Selon la littérature, il n'existe pas de prédilection sexuelle de la toxoplasmose oculaire. En ce qui concerne notre observation, la patiente est de sexe féminin, âgé de 72 ans, présentant une baisse de vision brusque, une grosse cicatrice choriorétinienne maculaire et d'ostéoblastes dans la région rétinienne périphérique de l’œil droit, typique de la rétinite pigmentaire à l'examen du fond d’œil. Ces signes illustrent les anomalies clinico-ophtalmologiques évocatrices de la toxoplasmose oculaire, et l’âge du patient constitue la particularité de notre observation. En effet, il y a peu ou presque pas d'observation de la toxoplasmose oculaire décrit chez la personne âgée, mais plusieurs auteurs [5–7] révèlent que l’âge moyen des patients souffrant de la toxoplasmose oculaire se situe entre 15 à 45 ans. Dans une étude réalisée au Brésil par Jones [8], la consommation de la viande insuffisamment cuite, manger de la viande salée, séchée ou fumée ou encore manger l'agneau congelé, travailler dans un jardin ou dans la cour plus d'une fois par semaine accroissait le risque d’être contaminé par Toxoplasma Gondii. Ce qui pourra être le cas avec notre observation qui relève la consommation exagérée de la viande. Par contre Balayre [9] a constaté que les personnes âgées peuvent être plus sensibles aux infections sévères de Toxoplasma Gondii en raison de la diminution de l'immunité à médiation cellulaire et la présence des maladies chroniques sous-jacentes. Par ailleurs, certains auteurs [10] signalent qu'en dehors du classique signe de toxoplasmose oculaire, d'autres pathologies peuvent être associées telles que la vascularite rétinienne, l'occlusion vasculaire rétinienne, le décollement de rétine rhegmatogène et séreuse, la sclérite et la rétinite pigmentaire unilatérale comme le cas dans notre observation. Selon la littérature, la confirmation du diagnostic de la toxoplasmose oculaire doit être basée sur les manifestations cliniques et les tests de sérologiques (IgM et IgG ainsi que leur avidité) [11]. D'autre part, la présence de la rétinite pigmentaire unilatérale classique dans la toxoplasmose oculaire chez une patiente de race noire, âgée de 72 ans a attiré notre attention. En générale, le traitement de la toxoplasmose oculaire demeure médical (antibiotique, corticoïde et antiparasitaire) [2, 12, 13].

## Conclusion

La toxoplasmose oculaire peut survenir même chez les sujets âgés de toute race. Son diagnostic est clinique, mais les tests d'anticorps de la toxoplasmose spécifique permettent de confirmer le diagnostic et de faire la différence entre la réactivation de l'infection et la toxoplasmose récemment acquise.
